# Tracing Scots Pine Expansion in Europe Using Patterns of Rare Alleles

**DOI:** 10.1002/ece3.72433

**Published:** 2025-11-16

**Authors:** Chedly Kastally, Jaakko S. Tyrmi, Catherine Bastien, María Teresa Cervera, Giovanni G. Vendramin, Outi Savolainen, Tanja Pyhäjärvi

**Affiliations:** ^1^ Department of Forest Sciences University of Helsinki Helsinki Finland; ^2^ Viikki Plant Science Center University of Helsinki Helsinki Finland; ^3^ Department of Ecology and Genetics University of Oulu Oulu Finland; ^4^ Biocenter Oulu University of Oulu Oulu Finland; ^5^ BIOFORA Unit INRA Orleans France; ^6^ Departamento de Ecología y Genética Forestal ICIFOR‐INIA‐CSIC Madrid Spain; ^7^ Institute of Biosciences and BioResources National Research Council Sesto Fiorentino Italy

**Keywords:** glacial refugia, *Pinus sylvestris*, range expansion, rare alleles, scots pine

## Abstract

Past climatic oscillations have likely played a key role in the evolution of all tree species in Europe, by causing alternating phases of range expansions and population declines. Nevertheless, tree species display remarkable variation in terms of spatial differentiation and levels of genetic diversity, suggesting that other factors have played an important part. Among wind‐pollinated trees, *Pinus sylvestris* displays an extremely low level of genetic structure over most of its distribution, but little is known about how this pattern has emerged. To better understand the processes that shaped this pattern of genetic diversity in *P. sylvestris* , we analyzed 11,020 SNPs from the nuclear and organellar genomes, sequenced from 185 trees across 20 populations from Europe, including the southern glacial refugia. We find that European populations as far west as France share most of their ancestry with southern populations from the Carpathian region. Populations from southwest Europe represent a second distinct gene pool, and populations from Italy a third. The variation in chloroplast and mitochondrial genomes highlights the importance of pollen dispersal in homogenizing genetic diversity over long distances. The population differentiation in the nuclear and organellar genomes is unlikely to be at drift migration equilibrium as suggested by our analysis of rare alleles. Finally, we found stronger and earlier demographic expansions in populations from the Carpathian region compared to Italy or southwest Europe. Overall, our study highlights the importance of topography and dispersal and the timing of demographic events in the evolution of a wind‐pollinated tree while illustrating the benefits of combining marker types and allele rarity to uncover fine levels of genetic structure.

## Graphical abstract

Strong and early demographic expansions from the Carpathian region shapes the current patterns of genetic diversity of *Pinus sylvestris* across Europe.

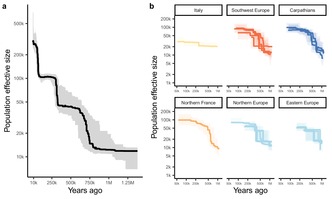

## 1 Introduction

Population expansions and declines are demographic processes that shape the genetic diversity of organisms with several consequences on their adaptation and evolution. For instance, the rapid fixation of alleles at the front of a range expansion wave can accelerate the adaptation of populations to new environments, or, adversely, can lead to the accumulation of deleterious alleles (Bosshard et al. 2019; Excoffier et al. 2009; Gilbert et al. 2020; Peischl et al. 2015; Zhang et al. 2016). Although these dynamics leave specific signatures on neutral genetic variation, complex population dynamics remain difficult to resolve, especially when several populations are involved. An important goal of evolutionary biology is to disentangle these dynamics as this helps us understand the history, evolution and adaptation of species.

In Europe, past climatic oscillations have caused alternating phases of forest contractions into isolated glacial refugia, when colder conditions gave rise to large glaciers, and phases of expansions in warmer conditions, once the glaciers shrank and melted (Hewitt 2000; Willis and van Andel 2004; Tzedakis et al. 2013). These cycles have left signatures on the distribution of genetic diversity in many European tree species, notably in glacial refugia, where populations survived and maintained old genetic variation (so‐called “hot spots” of genetic diversity) and in zones of secondary contact (or “melting pots”), where expanding populations from distinct refugia met (Petit et al. 2003). In several wind‐pollinated trees, such as *Fagus sylvatica, Picea abies* or *Populus tremula* , the genetic structure of European populations matches well the expected patterns of populations expanding from several isolated glacial refugia (Chen et al. 2019; Magri et al. 2006; Taberlet et al. 1998; Tollefsrud et al. 2008). In contrast, some tree species, such as *Pinus sylvestris* or *Betula pendula* , are genetically homogeneous across most of their European distribution (Bruxaux et al. 2024; Milesi et al. 2024; Dvornyk, et al. 2002; Pyhäjärvi et al. 2007; Salojärvi et al. 2017), suggesting that past climatic oscillations had a different or much weaker effect, or that other factors played a bigger role in shaping the current patterns of genetic diversity.

The case of *P. sylvestris* is arguably the most extreme known case, as it is the species that displays the lowest levels of population differentiation across much of Europe and at the same time shows the most extreme excess of rare alleles across several conifer and angiosperm trees (Milesi et al. 2024). Such extreme patterns do not seem to be explained by the biology of *P. sylvestris* , and they are in contrast with the genetic adaptive differentiation found along latitude (Mikola 1982; Kujala et al. 2017, 2023). These findings raise the question: what evolutionary processes could explain the patterns of genomic variation of *P. sylvestris* in Europe? Although several studies have explored its genomic variation, none have investigated patterns at the scale required to solve this question. Earlier studies have, for example, focused on historical processes at a deeper evolutionary time, such as the divergence between Asian and European populations (Bruxaux et al. 2024) or between species (Milesi et al. 2024; Zhao et al. 2024), or focused on other aspects, such as the adaptation of *P. sylvestris* to northern conditions (Tyrmi et al. 2020; Hall et al. 2021). Finally, none of these studies have collected genomic data from across all glacial refugia in Europe, and thus, critical information may be lacking.

In species with high dispersal abilities, such as *P. sylvestris* , alleles quickly spread across their range, making it difficult to uncover the history of populations at a fine resolution, but several approaches can improve the resolution of genomic inferences. Although a careful sampling design, including as many samples and loci as possible, is especially important, to make full use of the genomic data collected, other considerations can also be valuable. First, combining loci from organellar and nuclear genomes allows estimating separately the gene flow from pollen and seeds (Ennos 1994). Since pollen, transmitting the chloroplast genome of the next generation, is dispersed over larger distances than seeds, which transmit the nuclear and mitochondrial genome, the genetic diversity of organellar and nuclear genomes will be shaped differently by the same population dynamics, which may provide valuable insights. Second, methods focused on the genomic patterns of rare alleles can be powerful to assess genetic structure at a finer scale, since rare alleles are more likely to occur in specific parts of the distribution (Slatkin 1985; Hofer et al. 2009). Furthermore, rare alleles are more likely to stem from more recent mutations than common alleles and thus may give valuable insights about recent demographic or admixture events (Kimura and Ohta 1973; Slatkin 1985; Schiffels et al. 2016; Bergström et al. 2020).

A challenge to all genomic studies of conifer species is the large size of their genome and the high proportion of repetitive elements, paralogs or other duplicated areas (Nystedt et al. 2013; Neale et al. 2014), which can lead to the misidentification of polymorphic positions in the genome. To identify and exclude these errors, methods relying on the relative balance of genotype calls (the Hardy–Weinberg equilibrium within panmictic populations) and/or the read‐ratio balance in heterozygote genotypes (McKinney et al. 2017) can be used. These methods are most powerful for alleles with intermediate frequency, but they lack statistical power when examining low‐frequency alleles. This is problematic in species such as *P. sylvestris* , since a large fraction of its genetic variation is made up of rare alleles. A far more powerful approach to remove false variants relies on sequencing the megagametophyte tissue, a haploid tissue found in the seeds, in which any heterozygous genotype detected is, by definition, an error that can be excluded. Using megagametophyte samples has some disadvantages, however, as it requires the dissection of seeds, which is laborious, and halves the number of genomes sequenced.

In this study, we use *P. sylvestris* as a model species to investigate how analyzing rare alleles and using the combined power of organellar and nuclear genomes can help to uncover the fine genetic structure of a species with a particularly homogeneous distribution of genetic diversity. We combine these approaches with demographic analyses to shed new light on how past climatic oscillations may have led to distinct patterns of genetic diversity in *P. sylvestris* compared to other tree species. To this end, we leverage sequencing data published by Tyrmi et al. (2020) produced from megagametophyte tissue of *P. sylvestris* seeds, together with new sequencing data from eight populations. This pooled data offers both a suitable sampling of the European distribution of *P. sylvestris* , notably from glacial refugia in Spain, Italy and the Carpathian region, to explore the effects of past climatic oscillations, and benefits from sequencing data produced from megagametophyte tissue, which allows a careful curation of the sequencing data from a highly repetitive genome.

## 2 Material and Methods

### 2.1 Sampling, DNA Extraction and Sequencing

**FIGURE 1:**
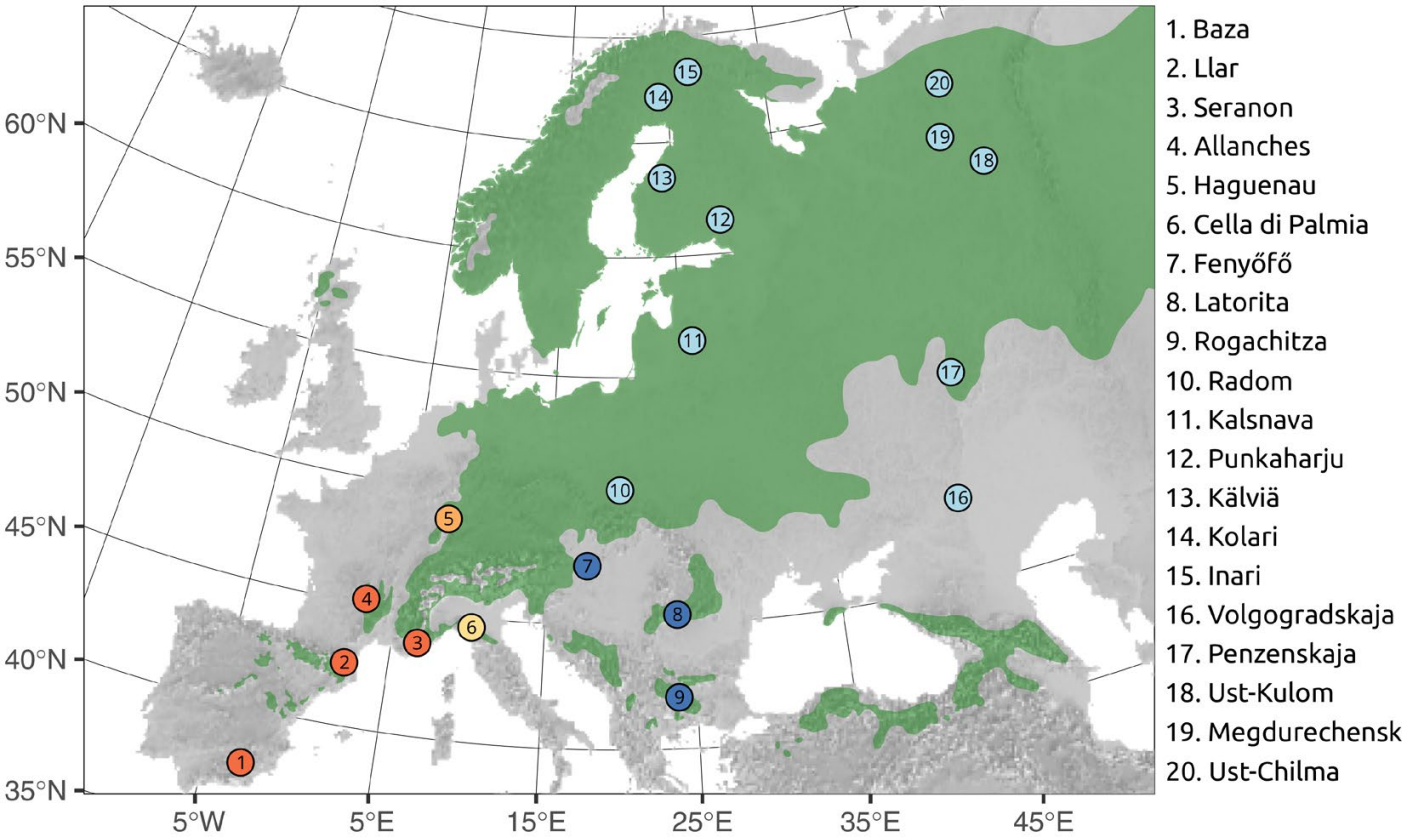
Sampling locations with colors indicating the grouping of the southern location into three pools (red: Southwest Europe; yellow: Italy; dark blue: Carpathians) and the remaining samples (orange: Northern France; light blue: Northern and eastern Europe). Physical map of Europe taken from www.naturalearthdata.com and the distribution (in dark green) of *P. sylvestris* from www.euforgen.org (Caudullo et al. 2017).

We collected needles of *Pinus sylvestris* from 66 individuals in 8 locations from southern Europe (Table 1; Figure 1). We targeted the three main areas identified as potential glacial refugia of *P. sylvestris* during the last glacial period in southern Europe (Cheddadi et al. 2006): southern France (Llar, Seranon and Allanches), Italy (Cella di Palmia), and the Carpathian region (Fenyőfő in Hungary, Rogachitza in Bulgaria and Latorita in Romania).

**TABLE 1:** Sampling location and summary statistics on genetic diversity for each studied population.

Population	Country	Sample size (ploidy^a^)	Latitude (°N)	Longitude (°E)	π_4_ (×10^−3^)	π_0/_π_4_	Tajima's D
Llar	France	8 (2)	42.5	2.2	2.38	0.37	−0.68
Seranon	France	8 (2)	43.8	6.7	2.40	0.35	−0.86
Allanches	France	6 (2)	45.2	2.9	2.35	0.34	−0.48
Haguenau	France	10 (2)	48.8	7.8	2.47	0.33	−1.02
Cella di Palmia	Italy	10 (2)	44.6	10.2	1.90	0.39	−0.26
Fenyőfő	Hungary	8 (2)	47.4	17.8	2.38	0.35	−0.75
Latorita	Romania	8 (2)	45.4	23.9	2.38	0.35	−0.70
Rogachitza	Bulgaria	8 (2)	42.1	23.8	2.37	0.34	−0.63
Baza*	Spain	8 (1)	37.8	−2.8	2.45	0.34	−0.20
Radom*	Poland	10 (1)	50.4	20.1	2.34	0.33	−0.45
Kalsnava*	Latvia	9 (1)	56.4	26.0	2.04	0.38	−0.26
Punkaharju*	Finland	18 (1)	61.5	29.2	2.11	0.33	−0.78
Kälviä*	Finland	10 (1)	63.4	24.1	2.32	0.34	−0.40
Kolari*	Finland	8 (1)	67.1	24.0	2.14	0.35	−0.26
Inari*	Finland	9 (1)	68.2	27.2	2.08	0.36	−0.30
Penzenskaja*	Russia	10 (1)	48.5	44.3	2.32	0.35	−0.17
Volgogradskaja*	Russia	9 (1)	53.5	46.1	2.27	0.35	−0.40
Ust‐Kulomsky*	Russia	8 (1)	61.5	54.0	1.95	0.37	−0.21
Evdino*	Russia	10 (1)	63.1	50.8	2.12	0.34	−0.24
Ust‐Chilma*	Russia	10 (1)	65.4	52.4	2.13	0.32	−0.38

*Note:* The nucleotide diversity was computed on 4‐fold and 0‐fold degenerate sites (π_4_ and π_0_), whereas Tajima's D was computed on 4‐fold degenerate positions. Populations marked with an asterisk were re‐analyzed from Tyrmi et al. (2020).

^a^ DNA extracted from megagametophyte tissue (haploid; 1) or needles (diploid; 2).

We extracted DNA using the E.Z.N.A. SP Plant DNA kit (Omega Biotek). Genomic DNA was fragmented with a Bioruptor ultrasonicator to a length of 200 nucleotides. Sequencing libraries were prepared using NEBNext DNA Library Prep Master Mix Set for Illumina and pools of four libraries using NEBNext Multiplex Oligos for Illumina E7600S (New England BioLabs). Targeted capture was performed for each pool according to the MycroArray MYbaits protocol v.2.3.1. (see Tyrmi et al. 2020).

In addition to these samples, we re‐examined 119 samples of *P. sylvestris* collected in 12 locations across southern, eastern and northern Europe (Tyrmi et al. 2020). These samples were sequenced using the same method, except that the DNA was extracted from the megagametophyte (haploid) tissue.

### 2.2 Read Mapping, Variant Calling and Quality Control

We aligned our sequences of *P. sylvestris* against the reference genome of the closely related *P. taeda* , combining its nuclear, chloroplast and mitochondrial genome assemblies together (Pita_v2.01, https://treegenesdb.org; and NC_021440.1 and NC_039746.1, available on Genbank). Reads were mapped using BWA v0.7.17 [*BWA‐MEM*; (Li 2013)], retaining only alignments with a mapping quality of at least 10 [samtools v1.9 “view ‐q 10”; (Li et al. 2009)]. Alignment files were cleaned and PCR duplicates removed using picard‐tools (v2.21.4; http://broadinstitute.github.io/picard) by using FixMateInformation, CleanSam and MarkDuplicatesWithMateCigar (default settings). We performed the variant calling using GATK [v4.1.4.0; (Van der Auwera and O'Connor 2020)] running successively the tools HaplotypeCaller (“‐‐emit‐ref‐confidence GVCF”), GenomicsDBImport, and GenotypesGVCF (“‐‐new‐qual”). To reduce the computational burden, we excluded regions with low coverage (less than an average of 5 reads per sample). We set the ploidy parameter to 2 and used the haploid samples to detect erroneous SNPs during the filtering steps. To accommodate for long running time, we ran the GenomicsDBImport and GenotypesGVCF steps for blocks of 1000 contigs at a time. All contigs were concatenated (bcftools v1.9 using tool concat; (Li et al. 2009)), and only bi‐allelic SNPs were retained (gatk SelectVariants ‐‐select‐type‐to‐include SNP). Additionally, we used the output files from the HaplotypeCaller (gvcf files) to estimate the portion of the genome we sequenced to discover SNPs.

### 2.3 SNP Filtering

Following GATK's best practice guidelines, we removed all positions with a quality depth ratio below 2, a mapping quality below 40, a strong strand bias (removing loci with either FS > 60 or SOR > 3.0), a score below −12.5 for the Rank Sum Test for mapping quality, a score below −8 for the rank sum test. In diploid samples, genotypes with a sequencing depth below 6 were set as missing, and we retained only sites with a maximum of 60% missing data. Additionally, we retained only SNPs that were called in at least 50% of the samples in all populations. The same criteria used to filter SNPs were used on monomorphic sites to estimate the size of the genome sequenced.

To exclude SNPs likely to be false calls, we scanned the genome to identify regions, or windows, enriched in erroneous SNPs detected using the following criteria: (1) heterozygous in a haploid sample, (2) heterozygosity level above 60%, (3) a read‐ratio deviation score D above 20 or below −20 (McKinney et al. 2017) and (4) when the Hardy–Weinberg Equilibrium test in diploid samples resulted in a *p*‐value below 0.05 in more than 3 populations out of 8. Finally, we computed the proportion of incorrectly called SNPs in windows of 250 bp and excluded all genomic regions with an error rate above 10%. These windows were used to exclude SNPs and were subtracted from our estimate of the size of the genome sequenced.

### 2.4 Variant Annotation, Pruning and Merging of Haploid and Diploid Samples

We used the script NewAnnotateRef.py (Williamson et al. 2014) to classify coding positions in the reference genome. We classified as neutral all SNPs falling in 4‐fold, intronic and intergenic positions or in positions not annotated in the reference. From the set of neutral SNPs, we identified SNPs in high linkage disequilibrium (LD) and randomly selected one SNP among pairs with *r* ^2^ above 0.8 within windows of 10 k bp. This was done separately for haploid and diploid samples. Finally, we harmonized the ploidy levels in our samples by splitting diploid samples in two by sorting alleles randomly, that is, assuming independence and linkage equilibrium across loci. Two data sets with haploid and diploid samples were thus obtained: one subset with all SNPs (subA) and one with SNPs pruned for LD (subP).

### 2.5 Genetic Diversity and Structure in *P. sylvestris*

To characterize the genetic diversity of *P. sylvestris* across Europe, we estimated the pairwise nucleotide diversity (π) on the basis of genetic variation at 0‐fold (π_0_) and 4‐fold (π_4_) positions (from the subA set) and the information from non‐variable sites extracted from the gvcf files produced by GATK. We estimated the ratio of π_0_/π_4_ to assess whether genetic load varied across populations, for example, between populations from glacial refugia and those outside.

To investigate the genetic structure of populations, we ran the following analyses on the pruned set of SNPs (subP). First, we performed a principal component analysis (PCA) with smartpca (eigensoft, using default settings; Patterson et al. 2006). We used Admixture to explore the clustering of our samples for *K* clusters from 2 to 6 (Alexander et al. 2009) and recorded the cross‐validation score at each value of *K* to identify which value maximized the model's accuracy. Finally, we used Arlequin (v3.5.2; Excoffier and Lischer 2010) to compute the pairwise *F*_st_ (Weir and Cockerham 1984) between all population pairs and to perform an AMOVA analysis.

### 2.6 Mitochondrial and Chloroplast DNA Analyses

We extracted SNPs identified on the mitochondrial and chloroplast genomes to characterize the genetic diversity and population structure of these genomes. Given the low coverage of organellar genomes we obtained, we adjusted the filtering criterion by relaxing the minimum sequencing depth to 1 but retaining only bi‐allelic SNPs with at most 25% of missing data for a locus, 25% of missing data for a sample and excluded loci where a heterozygous genotype was found. We then estimated the neighbor‐joining tree and haplotype networks for each organelle genome using R package *pegas* (v1.1; Paradis 2010) and used *igraph* (v1.3.1; Csardi and Nepusz 2006) and *ggnetwork* (*v0.5.12*; Briatte 2023) for their visual representation. Finally, we used the function *community_louvain* (in *igraph*), which implements an algorithm to uncover the community structure in a network (Blondel et al. 2008). We used a resolution of 0.2 to limit the number of clusters, and the edges of the network were weighted by the number of mutation(s) to reflect the genetic distances between haplotypes. We used Arlequin to estimate pairwise *F*_st_ between all population pairs.

### 2.7 Isolation by Distance and Estimation of Gene Flow

We investigated the patterns of isolation by distance (IBD) separately for each genome (nuclear, mitochondrial and chloroplast). First, using the R package “ape”, we tested whether genetic distances [*F*_st_/(1—*F*_st_)] and geographic distances (in km) were correlated using a Mantel test. Briefly, the test *p*‐value was obtained by comparing the observed correlation between the matrices of genetic and geographic distances and the distribution of 999 estimates after random permutation of the populations' location. Since we found evidence of IBD, we estimated the effective number of migrants (*Nm*) following Rousset (1997) as the inverse of the slope of the linear regression between genetic distance and the logarithm of the geographic distance. We further investigated whether the pattern of IBD changed when considering different subsets of the data. To this end, we repeated the same procedure focusing on the Carpathian, Italian or southwest Europe populations on gene flow by considering only pairs including one population from a focal glacial refugium and one from any other origin. We then compared the estimates of *Nm* obtained. We estimated the ratio of pollen to seed flow using the estimates of *F*_st_ for maternally, paternally and biparentally inherited loci using equations 5 and 6 from Ennos (1994).

### 2.8 Inferring the Origin of the Northern and Eastern Populations of *P. sylvestris*

We further wanted to investigate the ancestry of *P. sylvestris* in northern and eastern Europe, and specifically how they relate to populations from southern and central Europe. To this end, on the basis of our PCA and on previous studies on *P. sylvestris* (Cheddadi et al. 2006; Pyhäjärvi et al. 2007; Kujala and Savolainen 2012), we pooled samples into three groups, matching three known refugia: Southwest Europe, encompassing Spain and populations in southern France (Allanches, Llar, Seranon), the Carpathians (Carpathian: Fenyőfő, Latorita, Rogachitza) and Italy (Cella di Palmia). We then measured the shared ancestry of our samples with each of these groups using “EIGMIX” (Zheng and Weir 2016). This method relies on the eigen decomposition of genetic variance to estimate the shared ancestry between each sample and one or several reference sets, similarly to an Admixture analysis, but with the benefit of measuring the shared ancestry with a predefined set of reference populations.

### 2.9 Proportions of Shared Rare Alleles

Following an approach developed by Schiffels et al. (2016), we explored the proportion of shared alleles between populations, focusing on alleles that are rare in the source populations identified previously (Southwest Europe, Northern Italy and Carpathians). Assuming neutral mutations, rare alleles allow us to investigate the effects of recent processes on genetic structure since recent mutations are more likely to be rare. We first computed the minor allele count *c* of each locus in the reference set (all sources together) and their allele frequencies in each source. Then, for a given individual *i*, we considered all loci *l*_1_, *l*_2_, …, *l*_n_ for which individual *i* carried the minor allele. Using these, we computed the proportion of shared rare allele between *i* and source population *j* at allele count c (hereafter, the PSRA(*c*)_*i*,*j*_) using the following formula:

$$\text{PSRA}{\left(c\right)}_{i,j}=\frac{1}{n}*\sum_{l=1}^{n}\frac{f_{l,j}}{\sum_{s=1}^{3}f_{l,s}}$$

with *n* the number of loci *l* with minor allele count *c* (in the source population) and for which individual *i* carried the minor allele; *f*_*l,j*_ the allele frequency of locus *l* in source population *j*; and *f*_*l,s*_ the allele frequency of locus *l* in source population *s*.We thus obtained, for each individual, three proportions of shared alleles for all loci of a given minor allele count.

We considered only loci with values of c from 1 to 8 since at counts 9 and beyond the PSRA(*c*) for all sources were similar in value (1/3) for most samples. Finally, we also computed the population average and standard deviation of PSRA(c) over all c from 1 to 8.

### 2.10 Demographic Inference

To investigate the demographic dynamic of our populations, we first estimated the test statistic Tajima's D for each population on the SNPs on 4‐fold degenerate positions (set subA). Further, using a custom R script, we computed the folded site frequency spectrum (SFS) for each population and for the overall sample using the neutral sites (set subA). To correct for the effect of missing data on the SFS, we projected the allele frequencies to 50% of the initial sample size of the population using the hypergeometric distribution. We used the SFS obtained and our estimate of the portion of the genome sequenced to estimate the changes of effective population size (*Ne*) over time using Stairway plot v2 (Liu and Fu 2020) on the overall sample and each population separately.

## 3 Results

### 3.1 SNP Calling and Filtering

After alignment and filtering of mapped reads, we obtained an initial number of 4 M SNPs called. Of those, 1.13 M were bi‐allelic SNPs, with at most 75% of missing data. We used this initial set of SNPs to identify and remove errors in repetitive regions of the genome (see Section 2, SNP filtering). Specifically, we estimated an error rate in 250 bp windows on the basis of the number of (a) heterozygous calls in haploid samples, (b) loci with heterozygosity above 60% in diploid samples, (c) loci with a read‐ratio deviation above or below 20 and (d) loci with Hardy–Weinberg Equilibrium (HWE) deviation among diploid samples (*p*‐value < 0.05) in more than 3 populations out of 8. Among haploid samples, 8 samples had an excess of heterozygous positions compared to all other haploid samples, likely because of contamination by diploid tissue during the seed dissection prior to DNA extraction. For those (2 from Baza, Kolari, Punkaharju, and Ust Chilma), we replaced all their heterozygous positions with missing data, but retained loci that were heterozygous in one of those samples and not in others. Overall, we identified 652 k heterozygous positions in haploid samples, which we marked as errors. The excess in heterozygosity, read‐ratio deviation and HWE deviations in diploid populations identified a lower number of erroneous calls, 73 k SNPs. Combining these analyses, from the initial set of 1.13 M SNPs, we evaluated 31 k windows of 250 bp, of which we retained 15 k, including 108 k SNPs. Finally, we used the genome annotation to identify neutral positions (in intronic, intergenic, 4‐fold, or non‐annotated positions), 4‐fold positions and 0‐fold positions, and removed sites with less than 50% of call rate in any one population. We obtained 13 k SNPs in neutral positions, 478 in 4‐fold positions and 985 in 0‐fold positions. Processing the non‐variable positions in the same manner (for missing data and excluding windows with high call errors on variable positions), we estimated the genome length we used to discover SNPs to be 11 k bp for the 4‐fold positions, 50 k for the 0‐fold positions and 487 k for the neutral positions. For our analysis of genetic structure, we used the neutral and pruned SNPs in high LD (*r* ^2^ > 0.8; separately for haploid and diploid samples) and retained a total of 11,020 SNPs.

Regarding the mitochondrial and chloroplast genomes, we identified a total of 38 k raw SNPs. However, since the coverage of the mitochondrial genome was at low depth (average depth below 10×) we applied no filtering in terms of sequencing depth, but did apply stricter filtering for missing data compared to the nuclear genome. We first marked all heterozygous positions as missing, and then, after filtering, we retained 162 and 167 samples with less than 25% missing data and 717 and 269 bi‐allelic SNPs with less than 25% missing data, respectively, for the mitochondrial and chloroplast genomes.

### 3.2 Genetic Diversity and Structure

We found that genetic diversity across populations of *P. sylvestris* was low (π_4_ = 0.0022 on average, SD = 0.00017) compared to other European tree species (Chen et al. 2019; Milesi et al. 2024). The highest estimate of genetic diversity was found in the population of Haguenau (0.0025), which may be explained by its geographic location, that is in an area of secondary contact between several glacial refugia (i.e., a “melting pot”, as observed in several tree species by Petit et al. (2003)). The estimate was lowest in Cella di Palmia (Italy; 0.0019), potentially because of the relative isolation and modest presence of *P. sylvestris* in Italy. Across Europe, we observed a negative correlation between π_4_ and latitude (Spearman rank correlation, rho = −0.63, *p*‐value = 0.004) and between π_4_ and longitude (rho = −0.69, *p*‐value = 0.001). This could be explained by a more recent establishment of populations in northern and eastern Europe than in the south. The average ratio of π_0_ over π_4_ was 0.350 (SD = 0.018), in the range of previous figures obtained for *P. sylvestris* (Tyrmi et al. 2020; Hall et al. 2021).

**FIGURE 2:**
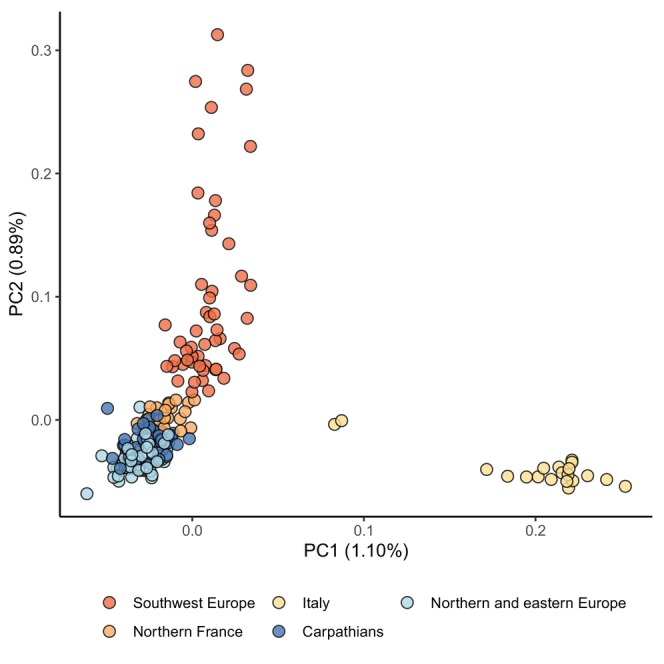
Principal component analysis with smartpca (Eigensoft). Circle coloring follows the scheme indicated in Figure 1.

**FIGURE 3:**
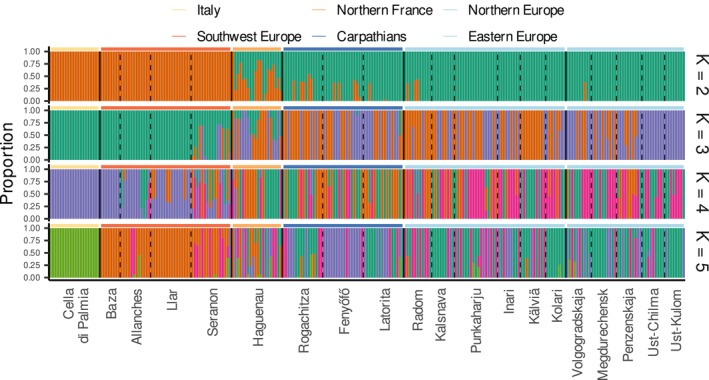
Admixture results for *K* values between 2 and 5. Thin vertical lines separate samples from separate locations (dashed) or regions (lines). Coloring of horizontal lines indicates the region of origin. The cross‐validation procedure found *K* = 1 maximized the model accuracy. However, with increasing *K*, a geographically consistent pattern emerges with three main genetic pools: (1) Italy, (2) Spain and southern France (Allanches, Llar, Seranon), and the (3) Carpathians (Fenyőfő, Latorita, Rogachitza).

Overall, the genetic differentiation between locations and regions was low, including the southern glacial refugia. On the basis of our AMOVA, 95.8% of the genetic variability is within population and 4.2% between populations (Table S1), supporting the pattern of a homogeneous genetic variation of *P. sylvestris* in Europe. Our principal component analysis (PCA; Figure 2) supports a genetic structure consistent with geography. Indeed, PC1 (1.15% of explained variance) captures the divergence of the Italian samples from all other samples, whereas PC2 (0.94%) separates the samples along a line going from the Spanish samples at one end to the samples from the Carpathian refugia at the other. Most samples, especially those from eastern and northern Europe, cluster with samples from the Carpathian region. Admixture results show that a model with one group explains best the genetic variation (retained *K* = 1), suggesting no or weak structure. Exploring the samples clustering for *K* between 2 and 5 (Figure 3) identifies largely the same patterns as those seen with the PCA: samples from northern and eastern samples mostly cluster with the samples from the Carpathian area, whereas the Italian and Iberian samples form a separate cluster. The Italian samples also appear as a separate cluster, although this pattern only emerges for higher values of K.

The highest pairwise *F*_st_ value was found between the Italian and Spanish populations (0.15; Table S2). Overall, the Italian population had the highest pairwise *F*_st_ values with other populations, with the Spanish population the second most diverged, with values ranging between and 0.02 and 0.06 with French populations and between 0.05 and 0.11 when compared to the populations from the Carpathian refugia and the populations in northern and eastern Europe. After Italy and Spain, the Ust‐Kulomsky population (Russia) had the highest pairwise *F*_st_ values, likely because of its extreme geographical location in our sampling.

**FIGURE 4:**
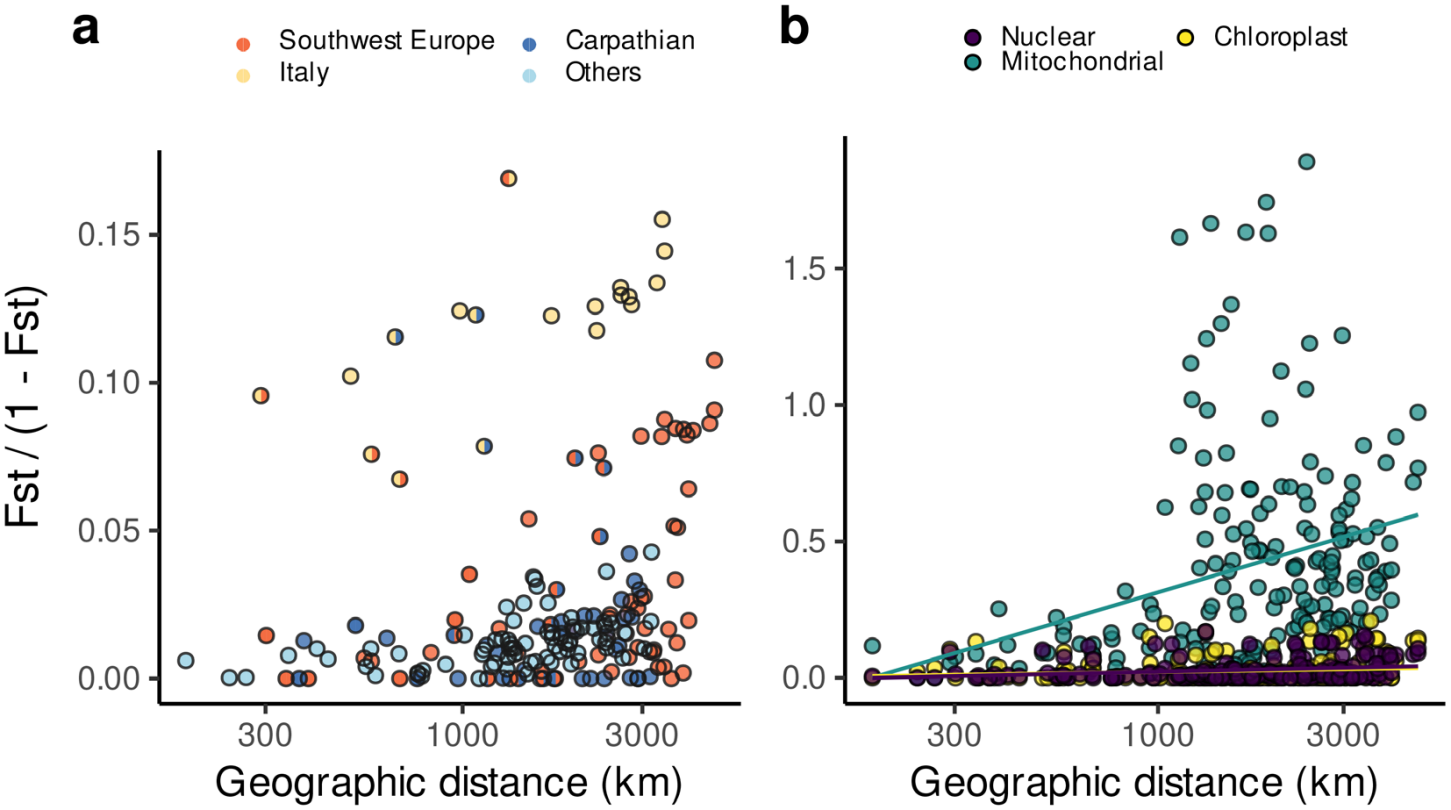
Correlation between the genetic distance (*F*_st_/[1—*F*_st_]) and geographic distance (km) (notice the logarithmic scale) estimated for each population pair (a) for the nuclear genome and (b) for nuclear, mitochondrial and chloroplast genomes. (a) Correlation between genetic and geographic distance for the nuclear genome. Circle colors identify the origin of the populations compared: Half circles represent pairs of populations from distinct glacial refugia, whereas full circles represent either pairs from the same refugium or pairs including at least one population from outside a glacial refugium. (b) The same correlation as in (a) but shown for the three genomes analyzed: Nuclear, mitochondrial and chloroplast (notice the difference of scales). The slope of the linear regression between genetic and geographic distances (lines) is inversely proportional to the effective number of migrants.

Finally, following Rousset (1997), we used the slope of the regression between geographic and genetic distance to estimate the effective number of migrants (*Nm*) between populations, which reflects in a stepping stone model the number of migrants per generation effectively contributing to the next generation. We found an estimate of 36, consistent with high levels of gene flow between populations of Europe. This value is high, but not surprising for a wind‐pollinated tree species. For instance, in other pine trees, values of *Nm* range from 3.4 in *P. edulis* (Premoli et al. 1994 in Ledig 1998) to 36.4 in *P. contorta* (Hamrick and Smith, unpublished, in Ledig 1998). Following the same approach, we estimated *Nm* separately for each glacial refugium. Interestingly, we observed distinct slopes (Figure 4a), reflecting the higher levels of divergence of our Italian population and the population from southwest Europe (*Nm* estimates of 23 and 48, respectively). These patterns are consistent with the higher geographical isolation of these refugia behind high mountain ranges, respectively the Alps and the Pyrenees. Removing the southwest European and Italian populations increases *Nm* to 56. These results are again consistent with the low levels of genetic differentiation of *P. sylvestris* populations in Europe, with the most divergent populations found in Italy and Spain (Kujala and Savolainen 2012; Tyrmi et al. 2020; Pyhäjärvi et al. 2008).

### 3.3 Pollen and Seed Gene Flow

**FIGURE 5:**
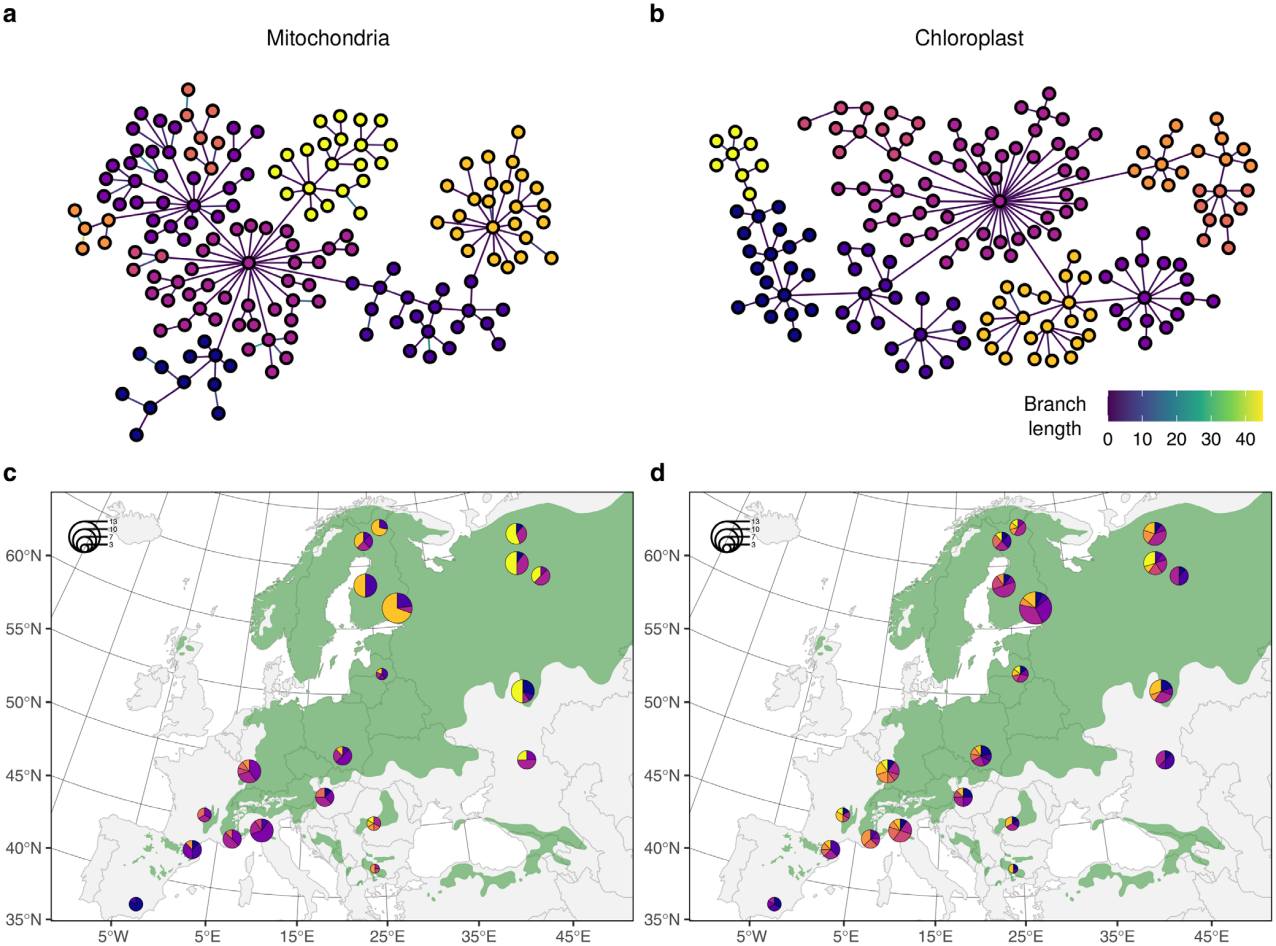
Distribution of the mitochondria (a, c) and chloroplast (b, d) haplotypic diversity across Europe in *P. sylvestris* . (a, b) Haplotype networks obtained from mitochondria (a) or chloroplast (b) genomes, including respectively 717 and 269 SNPs. Nodes in the networks represent haplotypes, and the branch coloring indicates the number of mutations between haplotypes. (c, d) Geographic origin of the mitochondrial (c) and chloroplast (d) haplotypes. Size of the pies reflects the number of samples, and the colors indicate the membership of each sample to clusters identified on the haplotype network (see Section 2). The distribution of *P. sylvestris* is indicated in dark green (Caudullo et al. 2017).

We characterized the population genetic structure on the basis of maternally and paternally inherited mitochondrial and chloroplast DNA. An interesting attribute of mitochondrial and chloroplastic loci is that, given that they are transmitted only by one parent, the mother or the father, the *Ne* for these loci is half of the nuclear loci. They are thus expected to have a more recent common ancestor than nuclear loci. Additionally, since, in conifers, chloroplasts are paternally inherited and dispersed through pollen and seeds, whereas mitochondria are maternally inherited and dispersed only through seeds, the mitochondria and chloroplast genomes can be used to estimate the ratio of gene flow transmitted by pollen and seeds (Ennos 1994; Petit et al. 2005). Out of the 717 and 269 retained SNPs, the majority were rare alleles, similar to the nuclear loci (we found 583 and 167 singletons, respectively, in the mitochondrial and chloroplast data). From these, we identified 162 and 167 haplotypes (all samples had a unique haplotype). The neighbor joining trees and haplotype networks for the mitochondria and chloroplast both reflected a stronger genetic structure in the mitochondria data (Figure 5), with a stronger divergence of populations geographically distant. Accordingly, the AMOVA results indicate that, respectively, 22% and 2% of the genetic variation is among populations in the mitochondria and chloroplasts. This is consistent with the fact that pollen disperses much longer distances than seeds.

We further found significant isolation by distance in our populations. Using Mantel tests, we obtained a significant association between genetic and geographic distances independently for all marker sets (two‐sided tests, *p*‐values = 0.007, 0.011, and 0.045, respectively, for the nuclear, mitochondrial and chloroplast sets, on the basis of 1000 permutations). Using our estimates of *F*_st_ for mitochondria, chloroplast and nuclear genomes, we estimated the relative rate of pollen and seed flow of 4.4 and 11.5, respectively, using equations 5 or 6 from Ennos (1994). Using the slope of the regression between genetic and geographic distance separately for mitochondrial and chloroplast loci gives estimates of *Nm* of 4 and 55, suggesting a consistent, albeit slightly higher, ratio of 13.8 between the two modes of dispersal (Figure 4b). Compared to previous estimates by Ennos (1994) of the ratio of pollen to seed flow in other tree species, *P. sylvestris* appears to have lower estimates than other tree species, which may be explained by higher seed dispersal.

### 3.4 Shared Ancestry With Populations From Glacial Refugia

To understand the history of *P. sylvestris* populations in Europe, we examined with Eigmix the proportion of shared ancestry of all populations against several source populations (Figure S1). We identified three sources on the basis of results on the genetic structure of *P. sylvestris* and from previous studies: (1) Italy (population Cella di Palmia); (2) Southwest Europe (populations Baza from Spain, and Llar, Seranon and Allanche from southern France) and (3) the Carpathians (populations Fenyőfő from Hungary, Latorita from Romania and Rogachitza from Bulgaria). Using Eigmix, we estimated the proportion of ancestry shared in individuals with each of the source populations. Eigmix relies on a decomposition of the eigenvector of a PCA, and we used the first 20 PCs derived by smartpca.

In this analysis, populations from northern and eastern Europe shared almost entirely their ancestry with those of the Carpathian populations. The ancestry of the Cella di Palmia population (Italy) was mostly independent from all other populations, although two Italian samples seemed to share a large fraction (35%) of ancestry with populations from Spain and/or southern France. Two of the southwest European populations had no or little sign of admixture, whereas two populations from southern France, Seranon and Llar, shared a significant proportion of ancestry with the Carpathian populations. Additionally, the population from Haguenau (northern France) also shared an important proportion of its ancestry with Carpathian samples, despite its closer proximity to samples from France and Spain. These results support the idea that the majority of European populations of *P. sylvestris* originate from the Carpathian, whereas the Italian populations remained isolated. Consistent with the PCA, there is a continuum of ancestry across populations of Europe, from the southwest to the central and eastern regions.

### 3.5 Proportions of Shared Rare Alleles

**FIGURE 6:**
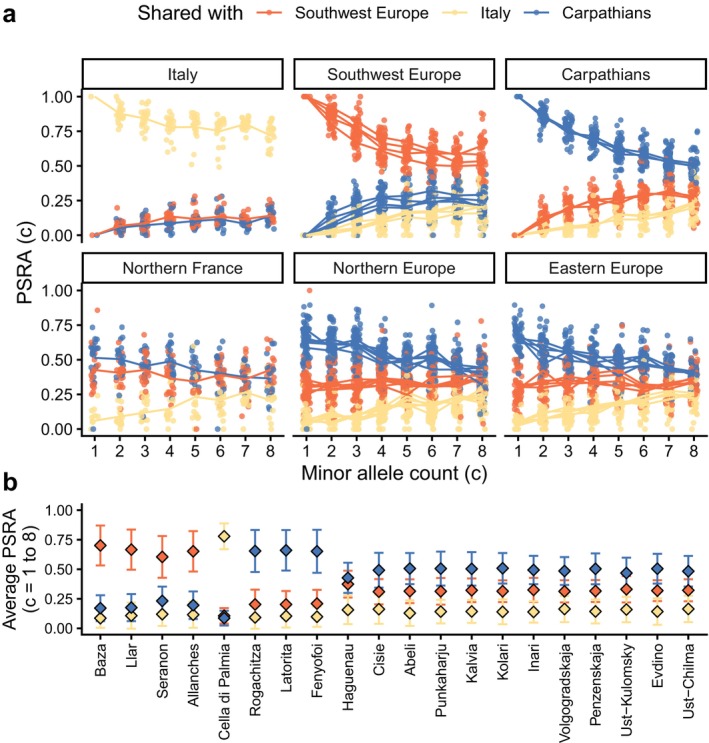
(a) Proportion of Shared Rare Alleles (PSRA) across minor allele counts *c* between each population and three source populations (Spain and southern France in orange; Italy in yellow; Carpathians in blue). Populations from different regions are plotted in separate panels (northern France: Hagenau; northern Europe: Radom, Kalsnava, Punkaharju, Kälviä, Kolari, Inari; eastern Europe: Penzenskaja, Volgogradskaja, Ust‐Kulomsky, Evdino, Ust‐Chilma; southwest Europe: Baza, Allanches, Llar, Seranon; Italy: Cella di Palmia; Carpathians: Fenyőfő, Latorita, Rogachitza). (b) PSRA averaged over allele counts 1–8 for all populations separately.

We further assessed the shared ancestry of our samples to each of the three potential sources but focusing on rare alleles. We limited our analyses to alleles with minor allele count below 9 in the source populations (10,571 SNPs) and measured the proportion of shared alleles between each individual and each one of the source populations, weighted by allele frequency (see Section 2). Finally, we computed an average and standard deviation at the population level of shared ancestry with each source. We observe a consistent pattern as with previous analyses in northern and eastern Europe: a high level of common ancestry with the Carpathian source, followed by the Southwest European source, and finally the Italian source (Figure 6a,b). Compared to our estimates obtained with EIGMIX, the ratio of ancestry from the Carpathian and Southwest Europe in populations from northern and central Europe was more balanced (closer to 1:1), with the population from Hagenau a ratio of 1.28, and in all other populations a ratio between 1.7 and 2.1 (except populations included in the refugia pools). This proportion of shared alleles fades for alleles with count 9 and above, for which no source has a greater proportion of shared alleles with any sample (above 9, all PSRA(c) converge to 1/3 for each source). The population of Haguenau (France) shares its alleles in a similar proportion with the Carpathian and Southwest Europe pools (Figure 6b), and this pattern of admixture displays little variation across allele counts (Figure 6a), contrary to other populations. This may suggest that the Southwest European pool shared with the Haguenau population more ancient mutations (i.e., influenced by more ancient processes) than those shared between Haguenau and the Carpathian pool. Population averages of PSRA(c) across minor allele counts 1–8 produced similar patterns (Figure 6b, Table S3), with most populations sharing the largest fraction of their alleles with the Carpathian pool. The average was homogeneous across populations from north and east Europe (around 0.5) but higher in the samples from the source populations themselves (at around 0.7).

### 3.6 Demographic Inference

**FIGURE 7:**
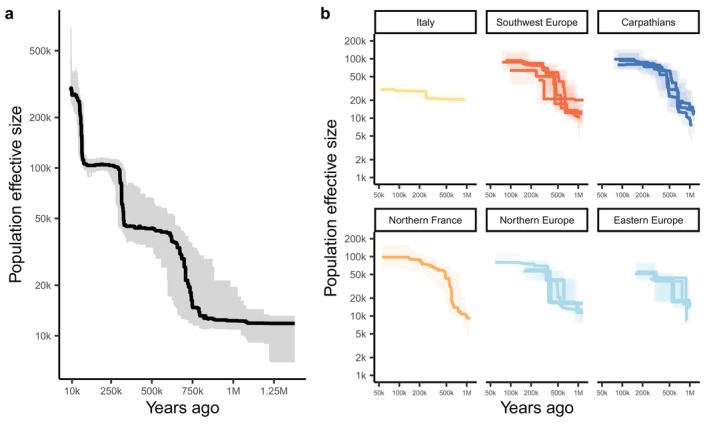
Changes in effective population size through time at (a) the species and (b) the population level inferred from the folded site frequency spectrum using Stairway plot 2. Solid lines represent the median estimates, whereas shaded areas indicate the 75% confidence intervals. We used a mutation rate of 15.9 × 10^−9^ per bp per generation and a generation time of 20 years.

To infer the demographic dynamic of our populations, we first estimated Tajima's D over 4‐fold positions (Table 1) using a custom R script. All our estimates were negative, indicating an excess of rare alleles in all populations, in line with estimates in previous studies. This is likely due to a demographic expansion across the *P. sylvestris* range (Pyhäjärvi et al. 2007; Kujala and Savolainen 2012; Bruxaux et al. 2024; Milesi et al. 2024). Interestingly Haguenau (France) had both the highest level of genetic diversity (as estimated by π_4_) and the most negative estimate of Tajima's D (−1.02), which reflects that the high genetic diversity in this population mainly consists of rare alleles, likely originating from diverse refugia. Our highest estimate of Tajima's D was found in eastern Europe, in Penzenskaja (Russia; −0.17), but relatively close estimates were found across Europe with no clear geographic pattern. Second, we used Stairway plot 2 to infer the past changes in *Ne* of *P. sylvestris* in Europe. We conducted this analysis at the species level and at the population level. At the species level (Figure 7a), we observed that *P. sylvestris* went through three distinct expansion events, first between 80 and 100 kya, then between 260 and 300 kya, and finally at around 550–750 kya. At the population level, we observe that all populations went through an expansion across a period going from 250 to 900kya. The population size increased by a factor of 6.3 on average (SD = 5.2), with the smallest values and most recent events obtained in populations from Cella di Palmia (Italy) and Baza (Spain) (1.5 and 2.2 fold increase, around 250 and 300 kya, respectively), whereas populations from the Carpathians experienced stronger demographic expansions (respectively, 6.9, 7.3 and 12.8 fold increase at 460, 480 and 650 kya). Likewise, the recent estimates of *Ne* are lower for the Italian and Spanish populations (30,980 and 43,818, respectively) than for populations in the Carpathians (*Ne* = 80,705, 93,090 and 98,282, respectively, for Hungary, Romania and Bulgaria). The fact that we do not detect multiple and especially recent demographic events in the single population analyses is likely due to the low power of the analysis when using smaller sample sizes.

## 4 Discussion

In this study, we examined the patterns of genomic variation of *Pinus sylvestris* in Europe to understand what contributed to its extremely low levels of genetic structure when compared to other European tree species. First, we found a weak but clear genetic differentiation between populations from the glacial refugia in southern Europe: southwest Europe, Italy, and the Carpathians. This is consistent with the expectations from the history of past climatic oscillations (Hewitt 2000; Petit et al. 2003). Specifically, across all our analyses, the Italian population appears as a particularly distinct pool, whereas we found a gradual differentiation between the Carpathian and southwest Europe populations, with one population from France standing out as an intermediate mix with elevated genetic diversity. This suggests that expansion fronts from separate glacial refugia, in Spain and in the Carpathians, have met in secondary contact zones, which seems to be limited to a narrow geographic space in western Europe in the case of *P. sylvestris* . At a broader scale, all populations from northern, central and eastern Europe group with the Carpathian populations, highlighting the historical importance of that single refugium in the evolution of *P. sylvestris* . This is in contrast to other species, where several refugia contributed to the postglacial expansion in Europe (Chen et al. 2019; Milesi et al. 2024).

Our demographic analyses provide a key insight into how the Carpathian populations of *P. sylvestris* may have come to expand predominantly in Europe. Indeed, although we found evidence of an increase in effective population size (*Ne*) in the past across all populations, the strongest and oldest signals of expansion in the glacial refugia were found in the Carpathian populations, 460kya to 650kya. By expanding at an earlier time, populations from the Carpathians may have benefited from a “first‐mover” advantage during the expansion of *P. sylvestris,* leading to a fairly uniform genetic composition of the species throughout most of Europe. This earlier expansion may have been favored by several factors. Populations from the Carpathians may have been better adapted to northern climatic conditions, compared to those from the Italian or Iberian peninsula. Furthermore, populations of *P. sylvestris* surviving in northern cryptic refugia in central and eastern Europe may have also contributed to their head start during the expansion phase of the species throughout Europe (Birks and Willis 2008). Another important factor is the European geography. Since the Pyrenees and the Alps are wider and higher mountains than the Carpathians, they have likely been a more important barrier to the northward expansion of forest trees. This is further compounded by the fact that the high level of local adaptation of populations of *P. sylvestris* may have limited their ability to overcome the climatic gradients found across elevation in a mountain range.

To further examine the relative contribution of each glacial refugia to the northward expansion of *P. sylvestris* populations, we analyzed the patterns of genomic variation using two additional approaches. Firstly, we looked at the patterns of isolation by distance (IBD) across Europe and at the patterns of pollen and seed dispersal. Overall, we find that pollen contributes largely to homogenizing the genetic diversity of *P. sylvestris* across large geographic distances, with a ratio of pollen to seed dispersal of 4–14, in the range of other pine species (Koski 1970; Ennos 1994). We found *F*_st_ in chloroplast SNPs (0.02), carried by pollen alone, 10 times lower compared to mitochondrial SNPs (0.20). The *F*_st_ in nuclear SNPs (0.04) is close to that of pollen, stressing the major effect of pollen dispersal on the genomic variation of nuclear DNA. Interestingly, when looking at the pattern of IBD in both organellar and nuclear genomes, we detect a pattern consistent with an isolating effect of the highest mountains, that is, the Pyrenees and the Alps, acting as geographical barriers to gene flow. In contrast, we detect no isolating effect of the Carpathians or of the Massif Central in France.

Secondly, we tested a novel method that separates the signal of genetic structure on allele count. This has the potential to outline temporal variations of gene flow (Hofer et al. 2009; Schiffels et al. 2016; Bergström et al. 2020). At the migration‐drift equilibrium, we would expect the proportions of shared rare alleles across populations to vary depending on the level of gene flow between them on the source populations (with lower gene flow leading to lower proportions of shared rare alleles). However, this is not the case here: overall, we observed a remarkable homogeneity of the proportion of shared rare alleles across tested populations and allele counts. A likely explanation is that populations of *P. sylvestris* are still far from the equilibrium and that the observed patterns close to panmixia at the continental level are unlikely to reflect current levels of gene flow between populations (Whitlock and McCauley 1999). This constitutes an important challenge for all genetic studies on the population structure of *P. sylvestris* , or other tree species, including ours. We also have clear evidence that *P. sylvestris* is far from the mutation‐drift equilibrium: our demographic analyses at the species level identify three expansion events in the last 750 k years, associated with a 15‐fold increase of the species‐wide *Ne*. This may compound further the challenge of detecting genetic structure across populations, since there is a low level of genomic variation to be found between isolated populations.

Although dating demographic events has a large degree of uncertainty and depends on several assumptions (the mutation rate and generation time, both difficult parameters to estimate), the expansions we detected in our analyses pre‐date the last glacial period, which started approximately 110 kya. Yet, it is unlikely that *P. sylvestris* populations have not experienced any population decline or expansion since then. It could be that the more recent events have been too weak relative to ancient demographic dynamics to be detected by our analyses. Additionally, a limitation of using the site frequency spectrum (SFS) to infer demographic changes is that complex and rapid population changes may potentially mask the effects of one another; for example, consecutive bottlenecks and expansions have opposite effects on the SFS and can thus be difficult to detect (Myers et al. 2008; Liu and Fu 2015). More powerful analyses using haplotypic information as well as larger sample sizes might be able to resolve the potentially more complex demography and population structure of *P. sylvestris* , but they require genomic resources that are still difficult to obtain in a species with such a large and repetitive genome.

A limitation of our study is that we focused on the European distribution of *P. sylvestris* , missing the effects of gene flow between our eastern samples and the population from Asia Minor or Asia. However, studies that included samples from Turkey (Pyhäjärvi et al. 2007) and/or populations from eastern Asia (Hall et al. 2021; Bruxaux et al. 2024) suggest these are genetically similar to European populations, despite the large geographic distances separating them. Therefore, it is unlikely that the patterns we have obtained would have been considerably different by including populations from these locations.

A major challenge when studying conifer genomes is the large proportion of repetitive areas in the genome, where misaligned reads lead to errors during variant calling. Typically, these errors can be filtered out by excluding genomic positions or regions with excessive ratios of heterozygous genotypes (e.g., above 0.6). However, this will only remove false variants that are widespread across the geographic distribution of the species, whereas rarer variants in the repetitive genome may not be as easily detected. This issue is not limited to conifer genomes; for instance, Jaegle et al. (2023) identified 3.3 M incorrectly called variants in *Arabidopsis thaliana* in a set of 1057 accessions because of cryptic copy number variation. This issue is likely to be also present in other genome studies on species with large and highly repetitive genomes and when using short‐read sequencing technologies. An excess of false variants due to repetitive regions should lead to both an inflated level of genetic diversity and a lower level of genetic structure. Here, we dealt with this issue by using a combination of approaches, for instance, using the haploid tissue in *P. sylvestris* seeds, the megagametophyte. Importantly, the number of false variants identified on the basis of the haploid tissue by far exceeded those detected on filtering the diploid tissue data alone.

In conclusion, our analysis of the demographic history of *P. sylvestris* in Europe highlights the dominant role played by a glacial refugium in the Carpathian region during the expansion of the species. The Italian, on the one hand, and the Spanish and the southern French populations, on the other, appear particularly diverged from populations in central, eastern and northern Europe. *P. sylvestris* displays a low level of genetic structure, and our results suggest that this is likely due to a combination of demographic and topographical conditions favoring the relatively earlier and faster expansion of a single refugium compared to others, and that this was possible because of the high dispersal capacity of this species.

## Author Contributions

**Chedly Kastally:** data curation (lead), formal analysis (lead), investigation (lead), methodology (lead), software (lead), visualization (lead), writing – original draft (lead), writing – review and editing (lead). **Jaakko S. Tyrmi:** data curation (equal), methodology (equal), validation (equal), writing – review and editing (equal). **Catherine Bastien:** conceptualization (equal), funding acquisition (equal), project administration (equal), resources (equal), writing – review and editing (equal). **María Teresa Cervera:** conceptualization (equal), funding acquisition (equal), project administration (equal), resources (equal), writing – review and editing (equal). **Giovanni G. Vendramin:** conceptualization (equal), funding acquisition (equal), project administration (equal), resources (equal), writing – review and editing (equal). **Outi Savolainen:** conceptualization (equal), funding acquisition (equal), investigation (equal), methodology (equal), project administration (equal), resources (equal), validation (equal), writing – review and editing (equal). **Tanja Pyhäjärvi:** conceptualization (equal), funding acquisition (equal), investigation (equal), project administration (equal), resources (equal), validation (equal), writing – review and editing (equal).

## Conflicts of Interest

The authors declare no conflicts of interest.

## Supporting information


**Appendix S1:** ece372433‐sup‐0001‐AppendixS1.pdf.

## Data Availability

Raw sequencing data are available from NCBI BioProject: PRJNA1091427. Code available from https://github.com/ckastall/pinus_sylvestris_europe_expansion.
