# Supplementary material for: Tracing Scots Pine Expansion in Europe Using Patterns of Rare Alleles
Source: Ecol Evol. 2025 Nov 16;15(11):e72433. doi: 10.1002/ece3.72433 (PMC12620029; doi:10.1002/ece3.72433)
Supplement: Supplementary file 1 — Appendix S1: ece372433‐sup‐0001‐AppendixS1.pdf. [file ECE3-15-e72433-s001.pdf]

Tracing Scots pine expansion in Europe using patterns of rare alleles

Chedly Kastally, Jaakko S. Tyrmi, Catherine Bastien, María Teresa Cervera, Giovanni G. Vendramin, Outi Savolainen, and Tanja Pyhäjärvi

Supplementary information

Table S1. AMOVA results on 11,020 SNPs performed with Arlequin v3.5.2.

Source of Variation	Sum of squares	Variance components	Percentage variation
**Among populations**	8646.0	13.3	4.2
**Within populations**	63714.8	302.9	95.8
**Total**	72360.8	316.2	100

Table S2. Pairwise F_{st} values between all populations. The most differentiated populations were Cella di Palmia (Italy), Baza (Spain) and, to a lesser extent, Ust-Kulom (Russia) in northeastern Europe.

	Llar	Seranon	Allanches	Cella di Palmia	Rogachitza	Latorita	Fenyőfő	Haguenau	Cisie	Kalsnava	Punkaharju	Kälviä	Kolari	Inari	Volgogradskaja	Penzenskaja	Ust-Kulom	Megdurechensk	Ust-Chilma
Baza	-0.02	0.03	0.02	0.14	0.05	0.07	0.07	0.05	0.07	0.08	0.08	0.08	0.08	0.08	0.08	0.08	0.10	0.08	0.08
Llar		-0.01	0.01	0.06	0.03	-0.02	-0.01	0.01	0.00	0.01	0.01	0.01	0.03	0.01	0.00	0.00	0.06	0.00	0.02
Seranon			-0.01	0.09	-0.00	0.01	0.01	0.01	0.02	0.02	0.02	0.02	0.03	0.03	0.02	0.02	0.05	0.02	0.03
Allanches				0.07	0.02	-0.02	-0.00	0.01	0.01	0.01	0.00	0.01	0.02	0.01	0.01	0.01	0.05	-0.00	0.01
Cella di Palmia					0.07	0.11	0.10	0.09	0.11	0.11	0.11	0.11	0.11	0.11	0.12	0.11	0.13	0.12	0.13
Rogachitza						-0.03	-0.02	0.00	-0.01	-0.02	-0.02	-0.01	0.00	-0.01	-0.01	-0.01	0.03	-0.01	0.00
Latorita							0.02	0.00	0.01	0.01	0.01	0.02	0.02	0.01	0.02	0.02	0.03	0.01	0.02
Fenyőfő								0.00	0.01	0.01	0.02	0.02	0.02	0.02	0.02	0.02	0.04	0.02	0.03
Haguenau									0.00	0.00	0.00	0.01	0.02	0.01	0.01	0.02	0.04	0.01	0.02
Cisie										0.00	0.01	0.01	0.01	0.01	0.01	0.01	0.03	0.01	0.02
Kalsnava											0.00	0.00	0.00	0.00	0.01	0.01	0.02	0.01	0.01
Punkaharju												0.01	0.01	0.00	0.01	0.01	0.02	0.01	0.01
Kälviä													0.01	0.00	0.02	0.02	0.03	0.01	0.02
Kolari														0.01	0.02	0.01	0.03	0.01	0.01
Inari															0.01	0.01	0.02	0.01	0.01
Volgogradskaja																0.01	0.03	0.01	0.02
Penzenskaja																	0.01	-0.00	0.01
Ust-Kulom																		0.00	0.01
Megdurechensk																			0.00

Table S3. The average and standard deviation of the Proportions of Shared Rare Alleles (PSRA) of each population and with each source population for alleles with minor allele counts from 1 to 8 in the source populations (Italy: Cella di Palmia; Southwest Europe: Baza, Allanches, Llar, Seranon; Carpathians: Fenyőfő, Latorita, Rogachitza). In bold the top PSRA for a given population. The ratio of shared alleles with the Carpathian over the Southwest Europe sources is also presented.

	**Italy**		**Southwest Europe**		**Carpathian**		**Carp. / Southwest E**	
Population	Average	SD	Average	SD	Average	SD	Average	SD
Baza	0.088	0.084	**0.746**	0.161	0.166	0.107	0.261	0.195
Llar	0.111	0.113	**0.717**	0.172	0.172	0.113	0.281	0.214
Seranon	0.127	0.105	**0.641**	0.188	0.232	0.122	0.443	0.316
Allanches	0.117	0.113	**0.686**	0.178	0.197	0.118	0.341	0.253
Cella di Palmia	**0.819**	0.110	0.100	0.063	0.081	0.060	0.895	0.539
Rogachitza	0.094	0.096	0.206	0.129	**0.700**	0.172	4.350	4.944
Latorita	0.105	0.098	0.207	0.118	**0.688**	0.175	3.576	2.786
Fenyőfő	0.102	0.089	0.211	0.117	**0.687**	0.180	3.768	3.927
Haguenau	0.166	0.128	0.393	0.117	**0.441**	0.132	1.275	0.705
Radom	0.166	0.126	0.320	0.108	**0.514**	0.156	1.915	1.225
Kalsnava	0.135	0.111	0.328	0.100	**0.537**	0.131	1.879	0.923
Punkaharju	0.150	0.106	0.321	0.115	**0.529**	0.143	2.105	1.807
Kälviä	0.144	0.098	0.336	0.109	**0.520**	0.145	1.913	1.402
Kolari	0.144	0.110	0.325	0.087	**0.531**	0.132	1.858	1.056
Inari	0.145	0.094	0.337	0.105	**0.518**	0.122	1.826	1.181
Volgogradskaja	0.169	0.113	0.328	0.099	**0.503**	0.123	1.750	0.860
Penzenskaja	0.149	0.102	0.325	0.097	**0.527**	0.131	1.883	1.040
Ust-Kulom	0.164	0.108	0.348	0.117	**0.489**	0.127	1.735	1.295
Megdurechensk	0.150	0.117	0.330	0.095	**0.520**	0.128	1.774	0.869
Ust-Chilma	0.170	0.114	0.335	0.102	**0.495**	0.132	1.780	1.587

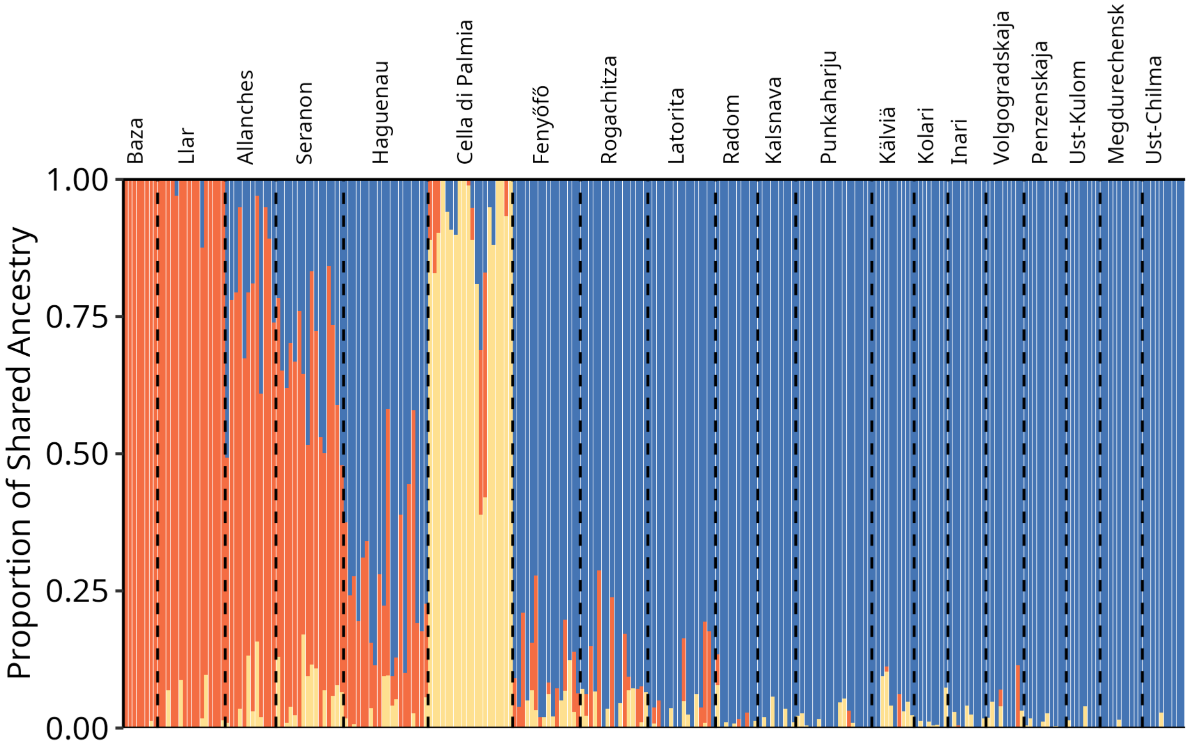

Figure S1. Proportions of shared ancestry in each sample with one of three source populations: Southwest Europe (orange; Baza, Llar, Allanches and Seranon), northern Italy (yellow; Cella di Palmia) and the Carpathian (blue; Fenyőfő, Rogachitza, Latorita). Results obtained using Eigmix.
